# Supplementary material for: TPPB modulates PKC activity to attenuate neuroinflammation and ameliorate experimental multiple sclerosis
Source: Front Cell Neurosci. 2024 May 22;18:1373557. doi: 10.3389/fncel.2024.1373557 (PMC11150779; doi:10.3389/fncel.2024.1373557)
Supplement: Supplementary file 1 [file Presentation_1.pdf]

Supplementary Figures

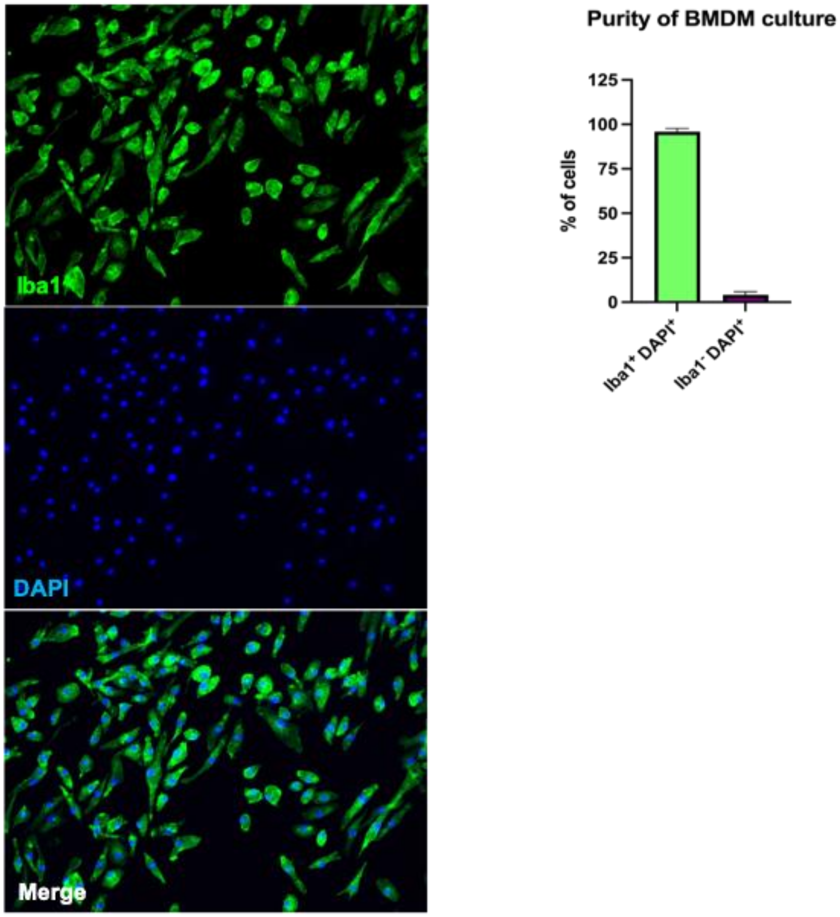

Supplementary Figure 1. Immunostaining analysis demonstrating the purity of isolated BMDM. The isolated BMDM cells were differentiated in the medium for 7 days, following which they were fixed in 4% PFA and stained for the macrophage marker Iba1 (green). Nuclei were counterstained with DAPI (blue). Representative images (*left*) from this experiment are shown. The bar graph (*right*) shows percentage of $Iba1^{+}DAPI^{+}$ and $Iba1^{-}DAPI^{+}$ cells. Over 95% cells were detected positive for Iba1 indicating that the overwhelming number of cells in the culture were macrophages.

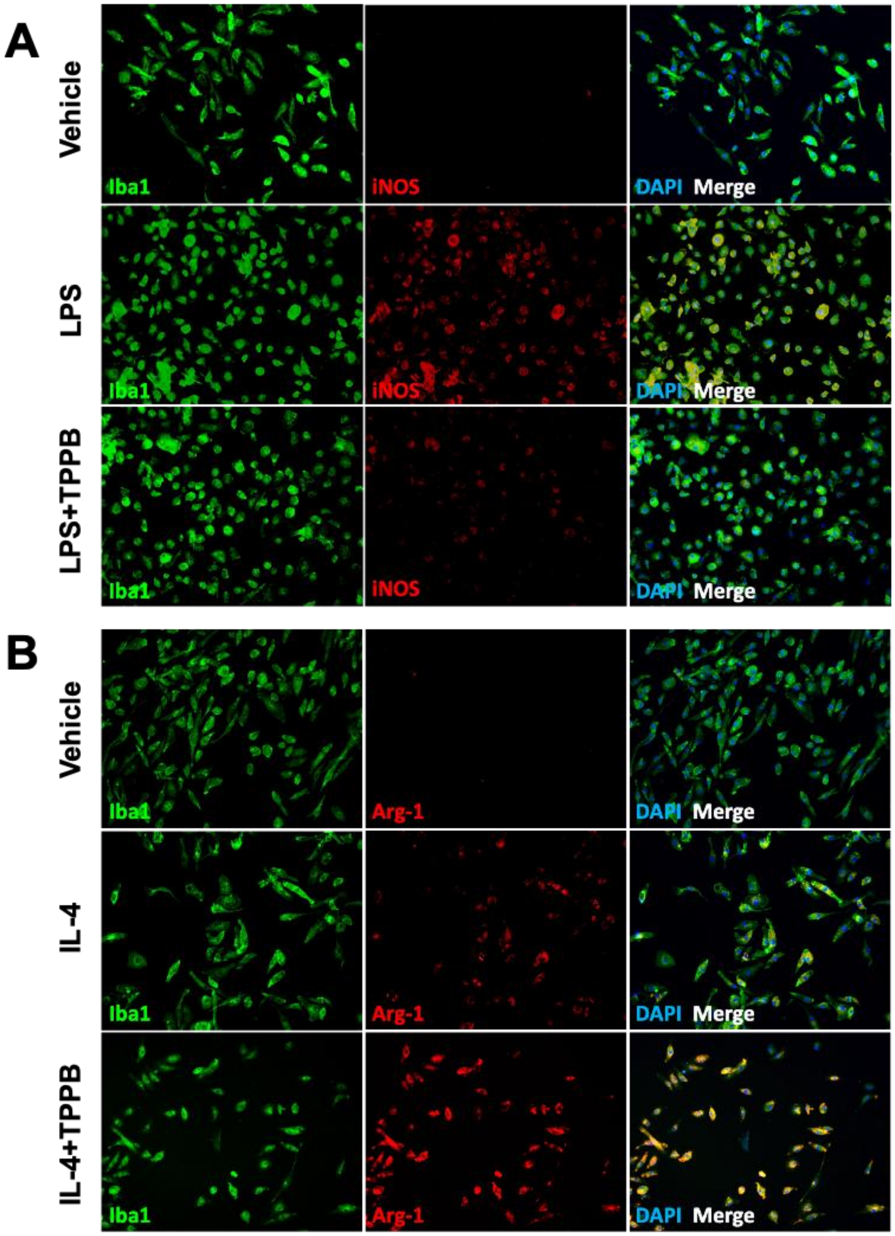

Supplementary Figure 2. Immunostaining of BMDM for pro-inflammatory and anti-inflammatory markers. Isolated BMDMs were treated LPS (100 ng/mL), IL-4 (20 ng/mL), and TPPB (100 nM) as indicated in the figure. (A) These cells were then stained for the macrophage marker Iba1 (green), iNOS (red), and DAPI (blue). (B) BMDM stained for Iba1 (green), Arg-1 (red), and DAPI (blue). Images are the representative of n=3.

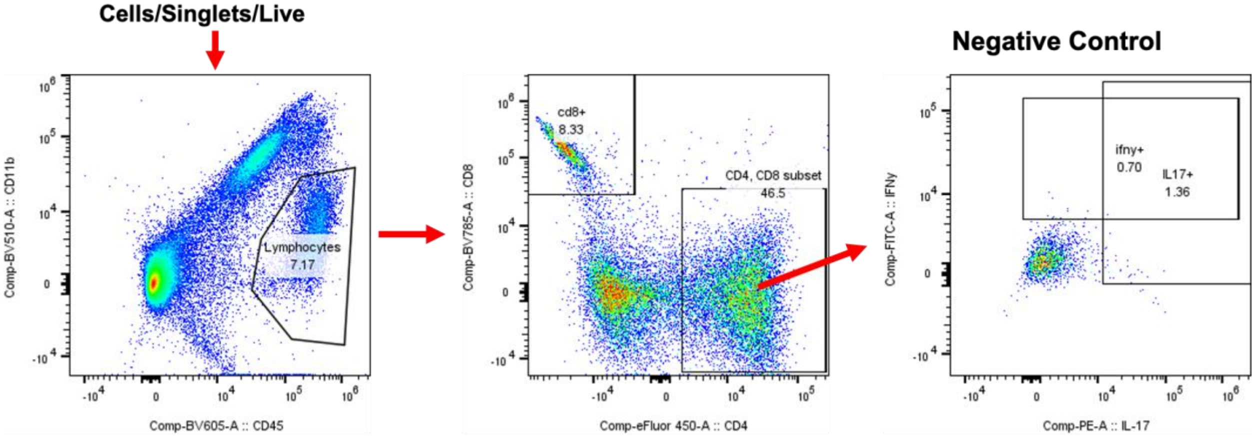

Supplementary Figure 3. Gating strategy and a representative negative control. Gating strategy for flow cytometry from spinal cords of $MOG_{35\text{-}55}$ EAE. After gating cells from FSC/SSC, removing doublets, and removing dead cells, lymphocytes were identified as $CD45^{+}CD11b^{-}$ cells. CD4 cells were then identified from this cell population, and the gating strategy for IL-17^{+} cells was determined by the negative control.

Supplementary Method

Immunofluorescence staining of BMDM

BMDM were grown on coverslips at a density $1x10^5$ cells/well in a 24-well plate. After the treatments, cells were fixed in 4% PFA for 10 min and washed twice with PBS. The cells were blocked with 2% BSA in PBS for 1 hr at RT following which they were incubated with primary goat anti-Iba1 (Novus Biological), rabbit anti-iNOS (Cell Signaling Technology), or rabbit anti-Arg-1 (Cell Signaling Technology) antibodies and diluted in blocking solution overnight at 4°C. After the incubation, the cells were washed thrice with PBS, and incubated with appropriate secondary antibodies for 1 hr in the dark at RT, followed by DAPI staining. Coverslips were then washed twice with PBS and mounted with VectaShield Plus Antifade Mounting Medium. Images were taken by Zeiss Axio Observer Z1 epifluorescence microscope at 20x magnification.
